# Optimized Extraction of Polysaccharides from *Bergenia* *emeiensis* Rhizome, Their Antioxidant Ability and Protection of Cells from Acrylamide-induced Cell Death

**DOI:** 10.3390/plants9080976

**Published:** 2020-07-31

**Authors:** Chen Zeng, Shiling Feng

**Affiliations:** College of Life Science, Sichuan Agricultural University, Ya’an 625014, China; zc@stu.sicau.edu.cn

**Keywords:** *Bergenia emeiensis*, polysaccharides, optimization, RSM, acrylamide-induced damage

## Abstract

*Bergeniaemeiensis* is a traditional herb in Chinese folk medicine. Most related studies are focused on the bioactivity of bergenin, neglecting other bioactive compounds. In our previous work, polysaccharides were identified in *B. emeiensis* rhizome. To evaluate the extraction process and the antioxidant ability of these polysaccharides, a response surface method and antioxidant assays were applied. The results showed that the yield of polysaccharides was highly affected by extraction time, followed by temperature and solvent-to-sample ratio. Under the optimal conditions (43 °C, 30 min and 21 mL/g), the yield was 158.34 ± 0.98 mg/g. After removing other impurities, the purity of the polysaccharides from *B. emeiensis* (PBE) was 95.97 ± 0.92%. The infrared spectrum showed that PBE had a typical polysaccharide structure. Further investigations exhibited the PBE could scavenge well DPPH and ABTS free radicals and chelate Fe^2+^, showing an excellent antioxidant capacity. In addition, PBE also enhanced the cell viability of HEK 239T and Hep G2 cells under acrylamide-exposure conditions, exhibiting great protection against the damage induced by acrylamide. Therefore, PBE can be considered a potential natural antioxidant candidate for use in the pharmaceutical industry as a health product.

## 1. Introduction

*Bergenia* emeiensis (*B. emeiensis*), belonging to the Saxifragaceae family, is a special species in China. In southwest China, *B. emeiensis* is found distributed at altitudes of 1300–1500 m. The plants of the genus *Bergenia* were well known in folk medicine for its beneficial effect on treating many diseases. In India, *Bergenia* was called Paashaanbhed and used for its hepatoprotective, diuretic and antipyretic properties. The aqueous extract of *Bergenia ligulata* rhizome could inhibit in vitro growth of CaC_2_O_4_ and calcium hydrogen phosphate dihydrate crystals [1]. Moreover, *Bergenia* plants were widely applied to treat diarrhea, vomiting, fever, cough, pulmonary infections, menorrhagia, excessive uterine hemorrhage, kidney stones and ulcer of large intestines [2,3,4]. Bergenin and arbutin were found to be the main polyphenol components in *Bergenia* [5]. Quercetin, kaemipferol and other flavonoids were also isolated from *Bergenia* [6]. Meanwhile, other flavonoids, anthraquinones and phenolic constituents were identified in some species of *Bergenia* [5,6,7,8]. However, analyses of the content and pharmacological activity of polysaccharides from *Bergenia* were rarely reported.

Polysaccharides from natural plants have been proven to possess a good biological activity. The polysaccharides from *Cyclocarya paliurus* showed great anti-hyperlipidemic and anti-diabetic ability [9]. Since the anticancer effect on polysaccharides has been proved, the antitumor ability of polysaccharides has gradually become a research hotspot [10]. In addition, natural polysaccharides could also effectively scavenge free radicals and increase the activity of antioxidant enzymes, thus exerting an excellent antioxidant capacity in vitro and in vivo [11,12]. 

Polysaccharides are condensed monosaccharide residues, containing a large number of hydrophilic groups with a high polarity. Thus, polysaccharides have good thermal stability and are easily soluble in water and insoluble in organic reagents such as petroleum ether, acetone, and ethanol. Therefore, the hot water extraction method is widely used to extract polysaccharides from natural plants with its advantages of mild extraction conditions, low equipment requirements and simple operation, but the method exhibits disadvantages such as high energy consumption, long time consumption and low efficiency. In addition to the extraction method, the extraction yield was also affected individually and interactively by extraction parameters such as extraction temperature, extraction time, solution pH and solvent to sample ratio. Therefore, a mathematical statistical technology—the response surface method (RSM)—was established to optimize the extraction process. Bb building a mathematical model based on a small number of experimental results, RSM has been widely applied to predict the optimal extraction process for its accurate estimation of the interactions between extraction parameters [13,14].

*B. emeiensis* was used as a folk medicine to treat respiratory diseases on the area of Mount Emei (Sichuan, China). However, the definite identity of the active compounds remains unknown. In this study, polysaccharides were considered as the index to investigate the content in *B. emeiensis* rhizomes. RSM was applied to optimize the extraction process. Moreover, the antioxidant ability of polysaccharides of *B. emeiensis* was evaluated by determined the scavenging ability on DPPH and ABTS free radicals and also the chelating capacity on Fe^2+^. The results may provide a theoretical basis for the potential application of the plant in the food, pharmaceuticals and cosmetics industries.

## 2. Results

### 2.1. Single-factor Experiment Analysis

**Figure 1:**
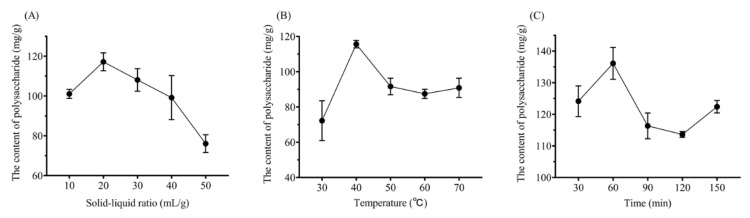
Effects of different extraction parameters on the yield of polysaccharides. (**A**) liquid-solid ratio, (**B**) extraction temperature and (**C**) extraction time.

To investigate the effect of a single extraction method on the yield of polysaccharides, single-factor experiments were carried out. When the liquid-solid ratio was changed from 10 to 20 mL/g, the yield of polysaccharides also increased. When the ratio was over 20 mL/g, the output decreased gradually (Figure 1A). Thereby, 20 mL/g was chosen as the center point for the RSM design. As shown in Figure 1B, the yield of polysaccharides reached the peak when the extraction temperature was 40 °C. The content tended to be stable as the temperature was increased over 40 °C. Hence, 40 °C was picked as the central point for the RSM design. Appropriately prolonging the extraction time could help the leaching of polysaccharides. As shown in Figure 1C, as the extraction time was extended from 30 to 60 min, the yield of polysaccharides was also promoted, but, over 60 min, the extraction rate declined and although the yield went up again at 150 min, it was still lower than that at 60 min. Therefore, 60 min was selected as the central point for the subsequent RSM design. 

### 2.2. Analysis of the Response Surface

Based on the outcomes of the single-factor assays, suitable extraction conditions were selected and used to design the RSM (Table 1). According to the experimental results and the multiple regression analysis, the yield of polysaccharides could be expressed using the following formula:

Y = 156.06 − 1.52A − 3.21B − 0.8744C − 3.48AB − 2.32AC − 2.05BC − 2.9A^2^ − 0.3684B^2^ − 3.35C^2^ (1)

where Y was the yield of polysaccharides; A was the extraction temperature; B was the extraction time and C was liquid-sold ratio.

The ANOVA analysis for the fitted quadratic model is shown in Table 2. The low p value of the model means the regression model was highly significant and a high p value of lack of fit indicated the model equation was suitable for predicting the yield of polysaccharides from *B. emeiensis*. A lower value of the coefficient of variation (0.6278%) meant the results were highly reliable. Moreover, the R^2^ was 0.9777 and adj R^2^ was 0.9491, both of which was close to 1, thus meaning the model exhibited a good accuracy and a high fitting degree for efficiently predicting the yield under different extraction conditions. In addition, as shown in Table 2, the p values of linear coefficients (A, B and C) were lower than 0.05. Interactions (AB, AC and BC) and quadratic terms (A^2^ and C^2^) were also significant (*p* < 0.05). Hence the relationship between three factors and the yield of polysaccharides was not simple linear relationship.

**Figure 2:**
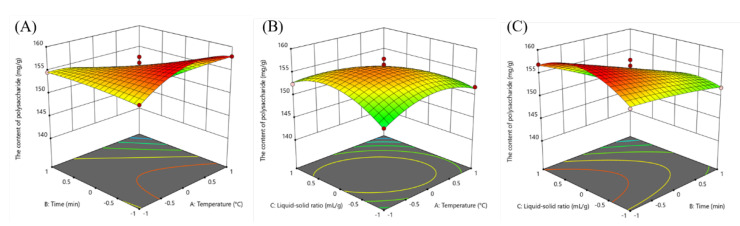
The response surface plots expressing the effect of extraction parameters on the yield of polysaccharides. (**A**) extraction temperature and extraction time; (**B**) extraction temperature and liquid-solid ratio; (**C**) extraction time and liquid-solid ratio.

A 3D surface map can intuitively reflect the interaction effects of factors in a binary regression model. As shown in Figure 2A, by fixing the liquid-solid ratio, appropriately increasing the extraction temperature could enhance the yield. When the temperature was fixed, the extraction rate increased as the extraction time decreased (Figure 2C). As shown in Figure 2B, fixing the extraction time, the yield was enhanced with the increase of the liquid-solid ratio and extraction temperature while the yield was decreased when the liquid-solid ratio and temperature were higher than the central point. Therefore, the interaction effects between two factors significantly affected the yield of polysaccharides.

Based on the RSM results the optimal extraction conditions were obtained: 43.11 °C, 30 min and 20.69 mL/g, which gave the highest yield of 159.25 mg/g. Considering the practical experiments, the extraction conditions were modified to 43 °C, 30 min and 21 mL/g. Under the modified conditions, the yield of polysaccharides was 158.34 ± 0.98 mg/g, which was close to the predicted value. Hence, RSM was successfully applied in this paper to optimize the extraction process.

### 2.3. Characteristics and Chemical Composition of Polysaccharides

**Figure 3:**
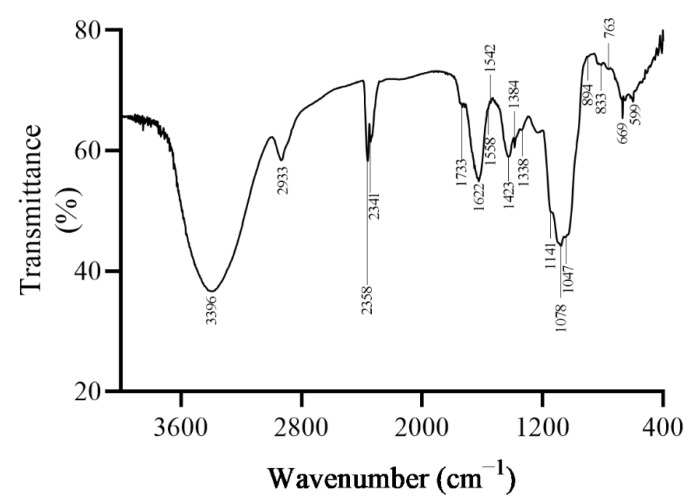
The FT-IR scanning spectrum of PBE.

To explore the primary structure of polysaccharides, the chemical composition and FT-IR scanning spectrum were determined. After purification, the content of polysaccharides from *B. emeiensis* (PBE) reached 95.97 ± 0.92% (Table 3). The protein that might be combined with PBE accounted for only 0.83 ± 0.025%. Therefore, we can state that most of the impurities were fully removed from the PBE. In addition, the molecular weight was 1.42 × 10^5^ Da according to a standard curve made using dextrans. As shown in Figure 3, a broad peak appeared at 3200–3400 cm^−1^ (3396 cm^−1^), that is attributed to the bending vibration absorption peak of the OH bonds caused by the stretching vibrations of the hydroxyl OH bonds related with the intermolecular hydrogen bonding [15]. A characteristic absorption peak of the polysaccharide substance ranging from 3000 to 2800 cm^−1^ (2933 cm^−1^) was the stretching vibration of the C-H bond, thus confirming that PBE was a mixture of polysaccharides. A strong absorption at 2300–2400 cm^−1^ indicated that the polysaccharides might contain CO_2_. In addition, the peaks between the range of 1300–1450 cm^−1^ showed the presence of -CH_3_ and -CH_2_ bending. The absorption peak at 1432 cm^−1^ was associated with the stretching and bending vibration of C-H bonds in the polysaccharides. Peaks at 1622 cm^−1^ and 1733 cm^−1^ were derived from carboxylate ion stretching bands and ester carbonyl groups, respectively [16]. Besides, the absorption peaks at 1000–1200 cm^−1^ (1078/1047 cm^−1^) corresponded to C-O-C stretching vibrations and proved that PBE had pyranose rings [17]. The absorption peak at 1542 cm^−1^ indicated that PBE contained other characteristic absorption peaks of polysaccharides (C-H and C-H_2_ stretching, C-OH bending vibrations) [18]. Many faint peaks in the region of 530–1000 cm^−1^ indicated the existence of β-glycosidic bonds with a pyranose ring (896.46 cm^−1^) [19].

### 2.4. Antioxidant Ability in vitro

**Figure 4:**
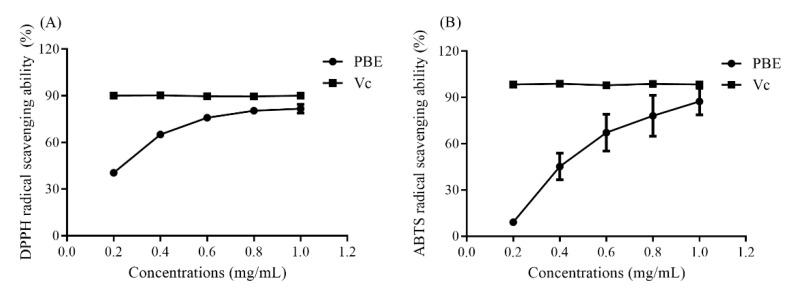

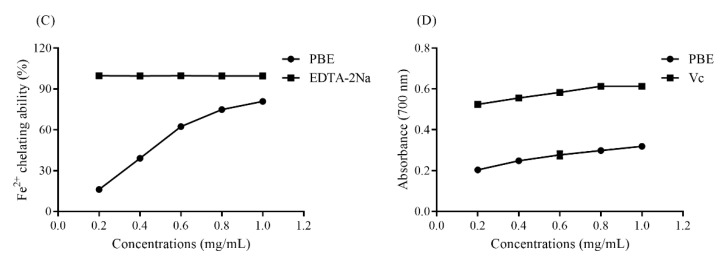
The antioxidant ability of PBE on (**A**) DPPH radical scavenging (**B**) ABTS radical clearance (**C**) Fe^2+^ chelating capacity and (**D**) the total reducing power.

Polysaccharides from plants were already proved to possess a strong antioxidant ability [20]. Polysaccharides from the fruit of *Rosa laevigata* had a noticeable effect on the radical scavenging of ABTS and DPPH [21]. In this study, scavenging on DPPH and ABTS radicals and total reducing power assays were conducted to investigate the antioxidant activity of PBE. As shown in Figure 4A, PBE could well clear DPPH at 1.0 mg/mL with a maximum clearance of 83.66%. Ascorbic acid (Vc) used as reference could scavenge 90.32% of DPPH radicals. When the concentration was higher than 0.6 mg/mL, the DPPH radical scavenging rate tended to be stable and close to that of Vc, with an EC_50_ of 0.31 mg/mL. As the concentration of PBE was increased, the ABTS radical clearance rate was also promoted, with an EC_50_ of 0.36 mg/mL (Figure 4B). Those two assays demonstrated that PBE possessed a strong free radical scavenging ability. Moreover, PBE also could chelate well Fe^2+^ (Figure 4C). The total reducing power of PBE also increased as the concentration increased (Figure 4D).

Polysaccharides from thirteen Boletus mushrooms were also proved to display a greater chelating activity with EC_50_ values ranging from 250.5 to 1413.8 μg/mL [22], and the EC_50_ of PBE was 0.48 mg/mL, which was higher than some natural polysaccharides, indicating PBE could become a potential antioxidant candidate.

### 2.5. Protection Against Acrylamide

**Figure 5:**
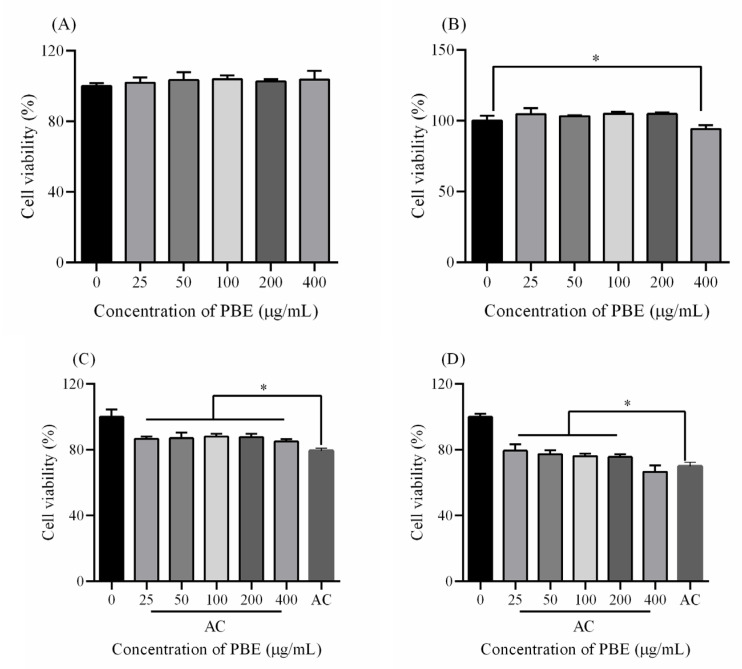
The toxicity of PBE on (**A**) Hep G2 cells and (**B**) HEK 293T cells. After treated with PBE for 24 h and exposed to AC for 10 h, the cell viability of (**C**) Hep G2 cells and (**D**) HEK 293T cells. Note: * means *p* < 0.05.

Acrylamide (AC) may cause potential harm to organs. Since *B. emeiensis* was used as a raw material for treating some injuries such as pulmonary infections and ulcers of the large intestine [2,3], to further explore the protective effects of PBE at a cellular level against the damage caused by AC, different concentrations of PBE were used to treat HEK 293T and Hep G2 cells. As shown in Figure 5A, none of the trial concentrations of PBE had a negative effect on the growth of Hep G2 cells, while the proliferation of HEK 293T cells was inhibited with 400 μg/mL of PBE (Figure 4B). When both kinds of cells were exposed to AC, the cell viability was reduced compared with the control group, but when pretreated with PBE for 24 h, the survival of Hep G2 was significantly increased (Figure 4C). Moreover, 0–200 μg/mL PBE also could enhance the cell viability of HEK 293T cell under AC exposure conditions. However, 400 μg/mL PBE suppressed the growth of HEK 293T, which may be related to a toxicity of high concentrations of drug (as 400 μg/mL PBE negatively affected HEK 293T) plus the toxicity of AC. Therefore, PBE showed a strong protective capacity for cells against the toxicity of AC, making PBE a promising alternative health product.

## 3. Discussion

Usually, a high temperature and a long extraction time would enhance the yield of polysaccharides obtained from many plants. Polysaccharides from the fruit of *Nitraria tangutorum* Bobr. was extracted at 60 °C for 7 h and the yield was 14.01% [23]. Polysaccharide from the wild mushroom *Paxillus involutus* was also extracted at 79 °C for 3 h to give the highest yield [24]. However, the extraction time for polysaccharides from flowers of *Dendrobium devonianum* was only 53.10 min and the polysaccharide exhibited an excellent antioxidant ability [25]. In this study, 40 °C and 30 min could make the output reach its highest value, indicating the polysaccharides from *B. emeiensis* could be distinguished from other polysaccharides and could possess a different bioactivity. The steep slope of the 3D graphs reflected the response sensitivity of the yield to the factors. A steeper 3D graph indicates a larger response value [26]. As shown in Figure 2, the three response surface plots were all steep and the contour maps were elliptic rather than a regular round shape meaning the interactions significantly influenced the yield. The results were exactly confirmed by the ANOVA analysis that the cross coefficients (AB, AC and BC) were significant (*p* < 0.05) (Table 3). Thereby, the RSM model were successfully established in this study to optimize the extraction process.

Reactive oxygen species (ROS) play an important role in maintaining the balance of homeostasis, signal transduction, and regulation of growth and development [27]. However, high concentrations of ROS could cause damage to proteins, lipids and nucleic acids, eventually leading to chronic diseases such as diabetes, atherosclerosis, cancer, and neurodegeneration [28]. Polysaccharides can not only directly bind to protons (H^+^) ionized by groups such as -OH and -COOH, but also chelate metal ions to prevent the generation of free radicals. Polysaccharides from purple sweet potato exhibited moderate DPPH radical scavenging activity and reducing power [29]. Polysaccharides from *Astragalus cicer* L. also could scavenge well ABTS and DPPH free radicals [30]. In this study, PBE could well clear DPPH and ABTS free radicals with the IC_50_ of 0.31 mg/mL and 0.36 mg/mL, respectively, exerting a higher antioxidant ability than other natural polysaccharides [31]. Maybe the extraction time and extraction temperature were lower than in other extraction conditions, which might prevent the -OH and -COOH from oxidation and even decomposition providing more functional groups meaning a great antioxidant ability [32]. Like other natural polysaccharides, PEB showed a strong free radical scavenging capacity, but there are few polysaccharides that could chelate metal ions. PBE also could chelate Fe^2+^, which was proved to a metal ion that can catalyze the Fenton reaction. Hence, the side outcomes such as lipid peroxidation would be prevented. 

Acrylamide (AC), listed as a category 2A carcinogen, was proved to cause damage to the human body [33,34]. Now, some natural plant extracts were found to relieve the toxicity caused by AC. Resveratrol could ameliorate the oxidative damage in rats under AC exposure conditions by activating the antioxidant system [35]. Curcumin also could lower the high ROS levels induced by AC in Hep G2 cells, and reduce DNA fragment formation, thus decreasing the toxicity of AC [36]. Moreover, AC causes damage to cells by increasing the ROS level then inducing oxidative injuries to cells and even causing death [33,35,36]. PBE showed a strong antioxidant ability to scavenge free radicals (Figure 3), hence, the protection of PBE on HEK 293T and Hep G2 cells against AC may be associated with the significantly free radical clearance since AC might cause oxidative stress to cells. However, more specific investigations shall be carried out to find out the mechanism.

## 4. Materials and Methods

### 4.1. Materials and Chemicals

*Bergenia emeiensis* was collected in the area of the Mount Emei (Sichuan, China) at an altitude of 1300–1500 m. The rhizomes were washed with distilled water and naturally air-dried. The rhizomes were ground into a fine powder, and kept at −20 °C for the following experiments.

2,2-Diphenyl-1-picrylhydrazyl (DPPH), was purchased from Sigma Chemical Co. (St. Louis, MO, USA). Ethanol, phenol, sulfuric acid, chloroform, butanol, trichloroacetic acid and glucose were purchased from the Chengdu Kelong Chemical Factory (Chengdu, China). FBS was obtained from Gibco (New York, NY, USA). Dulbecco’s modified eagle medium (DMEM), PBS and digestive enzyme were purchased from Hyclone (Logan, UT, USA). All chemicals were analytical grade.

### 4.2. Determination of Polysaccharides

The content of polysaccharides was determined by the phenol-sulfuric acid method [37]. Briefly, 100 μL phenol was mixed with 200 μL sample solution and 500 μL sulfuric acid. The reaction solution was standing for 30 min after shaking. Then the absorbance was measured at 490 nm. The standard curve was made with glucose.

### 4.3. Extraction Polysaccharides from Bergenia emeiensis

The powder of *B. emeiensis* was mixed with distilled water to different liquid-solid ratios (10, 20, 30, 40 and 50 mL/g) under various temperature (30, 40, 50, 60, and 70 °C) for 30, 60, 90, 120 and 150 min. Then the extract solutions were centrifuged for 10 min at a speed of 8000 r/min. The supernatant was collected to determine the content of polysaccharides.

### 4.4. Response Surface Design

Based on the outcomes of single factors assays, a three-level-three-factor Box-Behnken design with RSM was employed to optimize the extraction process. The yield of polysaccharides was taken as the response. The coded and actual levels of the three factors were presented in Table 4. Analysis of variance (ANOVA) was performed to detect the individual linear, quadratic and interaction regression coefficients using Design Expert 11 trial version (Stat-Ease, Inc., Minneapolis, MN, USA). The significance of the dependent variables was statistically analyzed by computing the *p* value.

### 4.5. Purification of Polysaccharides

The extract was filtered and concentrated using a rotary evaporator. An equivalent volume of trichloroacetic acid (20%) was added to the concentrate and the mixture was centrifuged at a speed of 5000 rpm for 5 min to remove the brown solids. Then ethanol was added to a final concentration of 85%. After standing at 4 ℃ for 24 h, the precipitate was separated and dissolved in distilled water. The solution was centrifuged again to remove impurities. Then supernatant was mixed 15 times with Savage reagent (chloroform:butanol = 4:1) to deproteinize it. The resulting aqueous solution was dialyzed for 3 days after removing the Savage reagent by using a rotary evaporator, and lyophilized to obtain polysaccharides (PBE).

### 4.6. Characterization of Purified Polysaccharides

#### 4.6.1. FT-IR Assay

5 mg of dry polysaccharides was milled with 500 mg KBr and then pressed onto a disc. The FT-IR spectrum of polysaccharides was identified using an infrared spectrometer (FT-IR-8400S, Shimadzu, Kyoto, Japan) in the range of 4000–400 cm^−1^.

#### 4.6.2. Determination of Protein

Protein content was measured by the Coomassie blue staining method [38]. Polysaccharides was dissolved in distilled water to 1.0 mg/mL. Coomassie brilliant blue G-250 (100 mg) was mixed with 50 mL 95% ethanol and 100 mL 85% phosphoric acid then diluted to 1 L with distilled water. Sample solution (0.1 mL) was mixed with 0.5 mL of Coomassie blue staining solution and 0.9 mL distilled water. The mixture was placed at 37 ℃ for 15 min in dark before reading the absorbance at 595 nm. The content of protein was calculated according to a standard curve made with bovine serum albumin.

#### 4.6.3. Determination of Molecular Weight

The molecular weight of polysaccharides was measured by gel permeation chromatography (Sephadex G100, 1 × 30 cm). Purified polysaccharides were resolved in distilled water to 10 mg/mL and loaded onto the chromatography column at 0.5 mL/min. The eluent was collected and determined the content of polysaccharides by phenol-sulfuric acid method to definite retention time. The standard curve was made with standard dextrans (T-10, T-40, T-70, T-100, T-500, and T-2000 series).

### 4.7. Determination of Antioxidant Ability

#### 4.7.1. Clearance Ability on DPPH Radicals

The scavenging ability on DPPH free radicals was conducted according to a previous method [39]. Briefly, 20 μL of different concentration of sample solution were mixed with 100 μL water and 80 μL DPPH ethanol solution (0.8 mM). Ascorbic acid (Vc) was used as the positive control. The reaction solution was placed at dark for 10 min before read the absorbance at 517 nm. The clearance rate was calculated according to the following formula:

Clearance rate (%) = 1 − A1/A0(2)

where A1 was the absorbance of sample group and A0 was the absorbance of the group with distilled water instead.

#### 4.7.2. Clearance Ability on ABTS Radical

The ABTS radical scavenging ability was tested according to a previous report with some modifications [40]. ABTS solution (7.4 mM) was equally mixed with potassium persulfate (2.6 mM) in the dark for 16 h. Then the mixture was diluted to an absorbance of 0.7. Sample solution (100 μL) was added to 100 μL of ABTS working fluid for 10 min before the absorbance of the reaction misture was read at 734 nm. The ABTS radical scavenging rate was determined by the following formula:

Clearance rate (%) = 1 − A1/A0(3)

where A1 was the absorbance of the sample group and A0 was the absorbance of the group with distilled water instead.

#### 4.7.3. Fe^2+^ Chelating Ability

The Fe^2+^ chelating ability assay was conducted according to a previous method with some modifications [22]. Briefly, 50 μL of sample solution was mixed with 100 μL of ferrous sulfate (0.00625 mM) and 50 μL of ferrozine (1 mM). The mixture was placed in the dark for 30 min before the absorbance was read at 562 nm with EDTA-2Na as the positive control. The chelating ability was calculated according to the following formula:

Chelating rate (%) = 1 − A1/A0(4)

where A1 was the absorbance of sample group and A0 was the absorbance of the group with distilled water instead.

#### 4.7.4. Total Reducing Power

The total reducing power was measured by q previous method [41]. Sample solution (0.2 mL) was mixed with 0.5 mL K_3_[Fe(CN_6_)] (1% *w/v*) and 0.5 mL phosphate buffer (0.2 M). The mixture was placed at 50 ℃ for 20 min then cooled at 0 °C after added 0.5 mL TCA (10% *w/v*). Subsequently, 0.1 mL aliquot of supernatant fluid was transferred into 1 mL distilled water and 0.1 mL FeCl_3_ (0.1% *w/v*). The absorbance of the reaction solution was read at 700 nm. An increasing absorbance meant an enhanced total reducing power.

### 4.8. The Protection Against Acrylamide

#### 4.8.1. The Cell Culture

HEK 293T and Hep G2 cells were kindly provided by the Biotechnology Center of Sichuan University (Sichuan, China). Cells were maintained in DMEM medium containing 10% FBS, at 37 °C in a 5% CO_2_ atmosphere. When the cell density was about 80% of the flask, the cells were digested from the bottle for following assay.

#### 4.8.2. The Toxicity of PBE on Cells

PBE was dissolved in PBS to different concentrations. Cell suspension (10^5^ cells/mL) was added into 96-well plate for 6. Then sample solution was transferred into the plate for 24. Subsequently, the solution was replaced with fresh medium containing 10% CCK-8 and placed at 37 °C for 30 min before the absorbance was read at 450 nm. The cell viability was calculated according to the kit instructions.

#### 4.8.3. The Remission on Acrylamide-induced Damage

Cell suspension was adjusted to 10^5^ cells/mL, then 90 μL solution was seeded into the plate for 6 h before 10 μL sample solution was transferred. After 24 h treatment, the cells were exposed to AC (6 mM) for 10 h, then the medium was replaced with new culture and CCK-8 and the plate was incubated at 37 °C for 30 min. The absorbance of each well was read at 450 nm then the cell viability was calculated.

### 4.9. Statistical Analyses

The data obtained in this study were analyzed statistically by ANOVA (GraphPad Prism 6, (GraphPad Software, Inc., La Jolla, CA, USA). Design-Expert 11 software (trial version, State-Ease Inc., Minneapolis, MN, USA) was applied to analyze the experimental results of the response surface design. All experimental results were expressed as mean ± standard deviation (SD). A value of *p* < 0.05 was considered to be significantly different.

## 5. Conclusions

In this work, a high content of polysaccharides as found in *Bergenia emeiensis* rhizome. The response surface method was successfully applied to optimize the extraction process. The optimal extraction temperature was 43 °C, the extraction time was 30 min and the solvent to sample ratio was 21 mL/g. Under these conditions, the highest yield was 158.34 ± 0.98 mg/g. After purification, the polysaccharides in PBE was 95.97 ± 0.92%, with a molecular weight of 1.42 × 10^5^ Da and containing only 0.83 ± 0.025% of protein. The FT-IR spectrum showed PBE contained a typical polysaccharide structure and also had pyranose rings. Moreover, PBE could well clear DPPH and ABTS free radicals and also chelate Fe^2+^ ion. Therefore, PBE possessed a strong antioxidant ability. Besides, PBE could also ameliorate and protect HEK 239T and Hep G2 cells from the damage induced by AC. More structural characterization of PBE should be conducted and also the antioxidant ability in cell level and in vivo need further investigation.

## Figures and Tables

**Table 1:** Box-Behnken experimental design and the results of these experiments.

Run	A-Temperature (°C)	B-Time (min)	C-Liquid-Solid Ratio (mL/g)	Y-Yield of Polysaccharides (mg/g)
1	40(0)	30(−1)	10(−1)	154.03
2	40(0)	90(1)	30(1)	146.55
3	40(0)	90(1)	10(−1)	151.91
4	40(0)	60(0)	20(0)	157.90
5	30(−1)	60(0)	10(−1)	150.05
6	30(−1)	60(0)	30(1)	152.44
7	40(0)	30(−1)	30(1)	156.89
8	50(1)	60(0)	10(−1)	151.82
9	50(1)	30(−1)	20(0)	157.98
10	30(−1)	30(−1)	20(0)	154.24
11	40(0)	60(0)	20(0)	155.55
12	40(0)	60(0)	20(0)	155.01
13	30(−1)	90(1)	20(0)	154.58
14	40(0)	60(0)	20(0)	155.20
15	50(1)	90(1)	20(0)	144.39
16	50(1)	60(0)	30(1)	144.95
17	40(0)	60(0)	20(0)	156.65
Experim.	43.11	30	20.69	159.25
Actual	43	30	21	158.34 ± 0.98

**Table 2:** The variance analysis of regression model.

Source	Sum of Squares	Mean Square	F-Value	*p*-Value
Model	283.37	31.49	34.15	<0.0001 ***
A	18.56	18.56	20.13	0.0028 **
B	82.57	82.57	89.55	<0.0001 ***
C	6.12	6.12	6.63	0.0367 *
AB	48.48	48.48	52.57	0.0002 **
AC	21.47	21.47	23.28	0.0019 **
BC	16.87	16.87	18.3	0.0037 **
A^2^	35.34	35.34	38.32	0.0004 **
B^2^	0.5713	0.5713	0.6196	0.457
C^2^	47.26	47.26	51.25	0.0002 **
Residual	6.45	0.9221		
Lack of Fit	0.6367	0.2122	0.1459	0.9271
R^2^	0.9777			
Adj R^2^	0.9491			
C.V. %	0.6278			

Notes: * means *p* < 0.05; ** means *p* < 0.01 and *** means *p* < 0.0001.

**Table 3:** The chemical composition of PBE.

Components	Content
Polysaccharides	95.97 ± 0.92%
Protein	0.83 ± 0.025%
Molecular weight	1.42 × 10^5^ Da

**Table 4:** Box-behnken design table of factors and levels.

Factors	Coding Level		
Extraction time	−1(30 min)	0(60 min)	1(90 min)
Extraction temperature	−1(30 °C)	0(40 °C)	1(50 °C)
Liquid-solid ratio	−1(10 mL/g)	0(20 mL/g)	1(30 mL/g)

## Sample Availability

Samples of *B. emeiensis* are available from the authors.

## References

[1] Joshi V.S., Parekh B.B., Joshi M.J., Vaidya A.D.B. (2005). Inhibition of the growth of urinary calcium hydrogen phosphate dihydrate crystals with aqueous extracts of Tribulus terrestris and Bergenia ligulata. Urol. Res..

[2] Ahmed E., Arshad M., Ahmad M., Saeed M., Ishaque M. (2004). Ethnopharmacological survey of some medicinally important plants of Galliyat areas of NWFP, Pakistan. Asian J. Plant Sci..

[3] Sinha S., Murugesan T., Maiti K., Gayen J.R., Pal B., Pal M., Saha B.P. (2001). Antibacterial activity of Bergenia ciliate rhizomes. Fitoterpia.

[4] Uniyal S.K., Singh K.N., Jamwal P., Lal B. (2006). Traditional use of medicinal plants among the tribal communities of Chhota Bhangal, Western Himalaya. J. Ethnobiol. Ethnomed..

[5] Chen X., Yoshida T., Hatano T., Fukushima M., Okuda T. (1987). Galloylarbutin and other polyphenols from Bergenia purpurascens. Phytochemistry.

[6] Zhao J., Liu J., Zhang X., Liu Z., Tsering T., Zhong Y., Nan P. (2006). Chemical composition of the volatiles of three wild Bergenia species from western China. Flavour Frag. J..

[7] Bohm B.A., Donevan L.S., Bhat U.G. (1986). Flavonoids of some species of Bergenia, francoa, parnassia and lepuropetalon. Biochem. Syst. Ecol..

[8] Yuldashev M.P., Batirov È.K., Malikov V.M. (1993). Anthraquinones of Bergenia hissarica. Chem. Nat. Compd..

[9] Mohib U.K., Muhammad N., Muhammad S., Shicong Z., Madiha R., Sundas F., Robina M., Yulin D., Rongji D. (2020). A review on structure, extraction, and biological activities of polysaccharides isolated from Cyclocarya paliurus (Batalin) Iljinskaja. Int. J. Biol. Macromol..

[10] Chen L., Huang G. (2018). Antitumor activity of polysaccharides: An Overview. Curr. Drug Targets.

[11] Wu G.H., Hu T., Li Z.Y., Huang Z.L., Jiang J.G. (2014). In vitro antioxidant activities of the polysaccharides from Pleurotus tuber-regium (fr.) sing. Food Chem..

[12] Liu Q., Zhu M., Geng X., Wang H., Tb T.B. (2017). Characterization of polysaccharides with antioxidant and hepatoprotective activities from the edible mushroom oudemansiella radicata. Molecules.

[13] Deniz B., Ismail H.B. (2007). Modeling and optimization I: Usability of response surface methodology. J. Food Eng..

[14] Said K.A.M., Amin M.A.M. (2015). Overview on the response surface methodology (RSM) in extraction processes. J. Appl. Sci. Process. Eng..

[15] Shi M.J., Wei X., Xu J., Chen B.J., Zhao D.Y., Cui S., Zhou T. (2017). Carboxymethylated degraded polysaccharides from Enteromorpha prolifera: Preparation and in vitro antioxidant activity. Food Chem..

[16] Guo Q., Cui S.W., Kang J., Ding H., Wang Q., Wang C. (2015). Non-starch polysaccharides from American ginseng: Physicochemical investigation and structural characterization. Food Hydrocolloid..

[17] Chen Y., Xue Y. (2018). Purification, chemical characterization and antioxidant activities of a novel polysaccharide from Auricularia polytricha. Int. J. Biol. Macromol..

[18] Su Y., Li L. (2019). Structural Characterization and antioxidant activity of polysaccharide from four Auriculariales. Carbohyd. Polym..

[19] Lin Y., Zeng H., Wang K., Lin H., Li P., Huang Y., Zhou S., Zhang W., Chen T., Fan H. (2019). Microwave-assisted aqueous two-phase extraction of diverse polysaccharides from Lentinus edodes: Process optimization, structure characterization and antioxidant activity. Int. J. Biol. Macromol..

[20] Kardošová A., Machová E. (2006). Antioxidant activity of medicinal plant polysaccharides. Fitoterapia.

[21] Liu X., Gao Y., Li D., Liu C., Jin M., Bian J., Lv M., Sun Y., Zhang L., Gao P. (2018). The neuroprotective and antioxidant profiles of selenium containing polysaccharides from the fruit of Rosa laevigata. Food Funct..

[22] Zhang L., Hu Y., Duan X., Tang T., Shen Y., Hu B., Liu A., Chen H., Li C., Liu Y. (2018). Characterization and antioxidant activities of polysaccharides from thirteen boletus mushrooms. Int. J. Biol. Macromol..

[23] Zhao B., Liu J., Chen X., Zhang J., Wang J. (2018). Purification, structure and anti-oxidation of polysaccharides from the fruit of Nitraria tangutorum Bobr. RSC Adv..

[24] Liu Y., Zhou Y., Liu M., Wang Q., Li Y. (2018). Extraction optimization, characterization, antioxidant and immunomodulatory activities of a novel polysaccharide from the wild mushroom Paxillus involutus. Int. J. Biol. Macromol..

[25] Wang D., Fan B., Wang Y., Zhang L., Wang F. (2018). Optimum extraction, characterization, and antioxidant activities of polysaccharides from flowers of Dendrobium devonianum. Int. J. Anal. Chem..

[26] Majeed M., Hussain A.I., Chatha S.A.S., Khosa M.K.K., Kamal G.M., Kamal M.A., Zhang X., Liu M. (2016). Optimization protocol for the extraction of antioxidant components from Origanum vulgare leaves using response surface methodology. Saudi J. Biol. Sci..

[27] Sauer H., Wartenberg M., Hescheler J. (2001). Reactive oxygen species as intracellular messengers during cell growth and differentiation. Cell. Physiol. Biochem..

[28] Vincent A.M., Russell J.W., Low P., Feldman E.L. (2004). Oxidative stress in the pathogenesis of diabetic neuropathy. Endocr. Rev..

[29] Sun J., Zhou B., Tang C., Gou Y., Chen H., Wang Y., Jin C., Liu J., Niu F., Kan J. (2018). Characterization, antioxidant activity and hepatoprotective effect of purple sweetpotato polysaccharides. Int. J. Biol. Macromol..

[30] Shang H., Wang M., Li R., Duan M., Wu H., Zhou H. (2018). Extraction condition optimization and effects of drying methods on physicochemical properties and antioxidant activities of polysaccharides from Astragalus Cicer L.. Sci. Rep..

[31] Rjeibi I., Feriani A., Hentati F., Hfaiedh N., Michaud P., Pierre G. (2019). Structural characterization of water-soluble polysaccharides from Nitraria retusa fruits and their antioxidant and hypolipidemic activities. Int. J. Biol. Macromol..

[32] Wang J., Hu S., Nie S., Yu Q., Xie M. (2016). Reviews on mechanisms of in vitro antioxidant activity of polysaccharides. Oxid. Med. Cell Longev..

[33] Yousef M.I., El-Demerdash F.M. (2006). Acrylamide-induced oxidative stress and biochemical perturbations in rats. Toxicology.

[34] Barber D.S., Hunt J.R., Ehrich M.F., Lehning E.J., LoPachin R.M. (2001). Metabolism, toxicokinetics and hemoglobin adduct formation in rats following subacute and subchronic acrylamide dosing. NeuroToxicology.

[35] Alturfan A.A., Tozan-Beceren A., Şehirli A.Ö., Demiralp E., Şener G., Omurtag G.Z. (2011). Resveratrol ameliorates oxidative DNA damage and protects against acrylamide-induced oxidative stress in rats. Mol. Biol. Rep..

[36] Cao J., Liu Y., Jia L., Jiang L.P., Geng C.Y., Yao X.F., Kong Y., Jiang B.N., Zhong L.F. (2008). Curcumin attenuates acrylamide-induced cytotoxicity and genotoxicity in HepG2 cells by ROS scavenging. J. Agr. Food Chem..

[37] Dubois M., Gilles K., Hamilton J.K., Rebers P.A., Smith F. (1951). A colorimetric method for the determination of sugars. Nature.

[38] Smith P.K., Krohn R.I., Hermanson G.T. (1985). Measurement of protein using bicinchoninic acid. Anal. Biochem..

[39] Brand W.W.M., Cuvelier M.E., Berset C. (1995). Use of a free radical method to evaluate antioxidant activity. LWT Food Sci. Technol..

[40] Xu G.Y., Liao A.M., Huang J.H., Zhang J.G., Thakur K., Wei Z.J. (2019). Evaluation of structural, functional, and anti-oxidant potential of differentially extracted polysaccharides from potatoes peels. Int. J. Biol. Macromol..

[41] Jia Z., Tang M., Wu J. (1999). The determination of flavonoid contents in mulberry and their scavenging effects on superoxide radicals. Food Chem..

